# Pythagorean Self Awareness Intervention in Caregivers of Patients with Motor Disabilities

**DOI:** 10.14806/ej.26.1.970

**Published:** 2021-08-23

**Authors:** Fotini Voulgari, Flora Bacopoulou, Artemios Artemiadis, Ioulia Kokka, Dimitrios Vlachakis, Xanthi Tigani, George P. Chrousos, Christina Darviri

**Affiliations:** 1 Postgraduate Course of Science of Stress and Health Promotion, School of Medicine, National and Kapodistrian University of Athens, Athens, Greece; 2 University Research Institute of Maternal and Child Health & Precision Medicine and UNESCO Chair on Adolescent Health Care, National and Kapodistrian University of Athens, Aghia Sophia Children’s Hospital, Athens, Greece; 3 Medical School, University of Cyprus, Nicosia, Cyprus; 4 Laboratory of Genetics, Department of Biotechnology, School of Applied Biology and Biotechnology, Agricultural University of Athens, Greece; 5 Lab of Molecular Endocrinology, Center of Clinical, Experimental Surgery and Translational Research, Biomedical Research Foundation of the Academy of Athens, Greece; 6 Department of Informatics, Faculty of Natural and Mathematical Sciences, King’s College London, Strand, London, United Kingdom

## Abstract

Caregiving for disabled people is a strenuous task often provided by family members, with adverse repercussions on the caregivers’ health. The aim of this study was to evaluate, for the first time, the effects of a novel cognitive-based stress management technique, the Pythagorean Self-Awareness Intervention (PSAI) on the stress levels and other cognitive and psychological characteristics of non-paid caregivers of patients with motor disability. In this quasi-experimental study, 59 caregivers of first-degree relatives with motor disabilities due to chronic neurological diseases, inpatients at a Rehabilitation Center, in Athens, Greece, were assigned to an intervention (PSAI, n=28) and a control group (received unstructured consultation, n=31). Psychological, cognitive, and sleep-related measurements (Zarit Burden Interview tool, Pittsburgh Sleep Quality Index, Self-Efficacy Scale, Depression, Anxiety and Stress Scale, Symbol Digit Modalities Test, California Verbal Learning Test-II, Brief Visuospatial Memory Test-Revised) were held at baseline and after 8 weeks (at completion of PSAI) in both groups. PSAI was found to decrease caregivers’ stress, depressive symptoms and anxiety and improve their sleep quality, visual memory, self-efficacy, and cognitive speed processing. Future randomized controlled studies are needed to investigate the effects of this novel intervention in larger samples of caregivers.

## Introduction

Disability can be defined as the reduced functional capacity due to mobility-related or other problems deriving from chronic illness or advanced age. Caregiving for disabled people is a strenuous and complex task which is often provided by family members, and has several repercussions (Roth et al., 2015). Caregivers may experience high levels of distress in the four aspects of their life; physical (Portenoy et al, 1994), spiritual, social and psychological (Carlson et al, 2004; Carlson and Bultz, 2003). Caregivers’ stress might be as high as it is for the patients themselves (Nijboer et al, 1998). More specifically, increased and/or long-term stress caused by caregiving has been linked to impaired social life, work-related problems, depression, chronic anxiety, cognitive problems, sleep disturbances, cardiac and metabolic diseases and even increased mortality (Lwi et al, 2017; Roth et al, 2015). These consequences are caused either directly by the hyperactivity of the stress system, or indirectly by the unhealthy lifestyle and social behaviours ascribed to the stress of caregiving (Allen et al, 2017). Stress-management seems to alleviate the adverse outcomes related to caregiving, as shown by two meta-analyses (Dharmawardene et al, 2016; Gilhooly et al, 2016).

The Pythagorean Self-Awareness intervention (PSAI) is a novel cognitive intervention based on 71 moral values (Golden Verses) described by the ancient philosopher Pythagoras (Guthrie, 1962). The PSAI incorporates these Pythagorean values to promote individuals’ self-awareness by changing the way they view others and themselves. The technique consists of three basic stages. On the first stage, individuals are called to sequentially recall daily events; on the second stage, to contemplate on thoughts and emotions derived from the recalled events; on the third and last stage, to critically appraise their attitude as internal observers. The difference of the PSAI from other relaxation techniques is the lack of focusing on objects, breathing or senses. For example, the Pythagorean Self-Awareness technique requires critical self-evaluation compared to the non-judgmental approach of mindfulness (Creswell, 2017). This difference encourages individuals practicing the technique to evaluate and modify their lifestyle, the way they connect with others and their behaviour. Via Pythagorean Self-Awareness, thought process and feelings interact and coordinate. To activate the internal observer, the individual must focus on inner self, concentrate, and mobilize the memory. On a neurobiological level, the key point of the technique is the Default Mode Network (DMN), a neural brain connection system related to the processes of cognition (Buckner et al., 2008). The role of DMN is significant in meta-cognitive processes and in adjusting individual behaviour via impulse’s inhibition (Shapira-Lichter et al., 2013). Through the three stages of the Pythagorean Self-Awareness technique individuals identify wrong choices and unhealthy behaviours and are motivated to change. This internal dialogue promotes a healthier lifestyle and may reduce stress and anxiety. The PSAI has been implemented in different populations, with positive results so far. The technique has been applied to obese, breast cancer and multiple sclerosis patients, as well as females with acne vulgaris (Anagnostouli et al., 2018; Charalampopoulou et al., 2020; Chatzikonstantinou et al., 2019; Simos et al., 2019). All studies demonstrated significant improvements of participants’ subjective stress levels, quality of life, cognitive tests, and other psychosocial characteristics.

The aim of this study was to evaluate, for the first time, the effect of PSAI on the stress levels and other cognitive and psychological characteristics of caregivers of inpatients with motor disability due to chronic neurological diseases. The initial hypothesis was that a holistic program of cognitive-based stress management such as the PSAI would improve the psychological dimensions, sleep quality and cognitive aspects of caregivers.

## Materials, Methodologies and Techniques

### Study design and participants

This non-randomized two-armed study was conducted at the Filoktitis Rehabilitation Center, in Athens, Greece. Study participants were recruited over a period of 6 months and were eligible for inclusion if they were i) 18 years or older, ii) caregivers of first-degree relatives of patients with motor disability due to chronic neurological diseases, iii) not receiving remuneration for caregiving, iv) providing care for at least 1 hour per day over at least one year, and v) able to read and write in Greek. Caregivers with a history of major psychiatric disease (*i.e.* depression, drug abuse etc.), under any type of psychotherapy, practicing any type of relaxation techniques, or having experienced a major life event within the previous 6 months, were excluded. Participants were allocated to either the PSAI group or the control group that received unstructured consultation. Assignment was made in a non-randomized fashion due to the time required to take part in the PSAI; considering the burden of responsibility a caregiver carries and the shortage of free time, the researchers decided to ask which caregivers were able to participate in the PSAI group, and thereby, the non-randomized fashion can be justified.

### Ethical considerations

The study protocol was approved by the Center’s Ethics Committee and was consistent with the Declaration of Helsinki. All participants were informed about the purpose of the study and provided written, signed consent prior to study entry. Participants were reassured that they could withdraw from the study at any stage without any consequences.

### Measures

#### Sociodemographic characteristics:

Participants had to complete a form regarding their sex, age, educational level, and family status.

The following psychological, cognitive, and sleep-related measurements were held at baseline and after 8 weeks (at completion of PSAI).

#### Zarit Burden Interview tool:

This is a 29-item caregiver self-report measure of burden. Each item on the interview is a statement which the caregiver is asked to endorse using a 5-point scale. Response options range from 0 (never) to 4 (nearly always) (Zarit et al., 1980).

#### Pittsburgh Sleep Quality Index (PSQI):

This is a 19-item self-report questionnaire that assesses the quality of sleep over a 1-month time interval. PSQI measures several aspects of sleep, with seven component scores and one composite score. The component scores consist of subjective sleep quality, sleep latency (*i.e*., how long it takes to fall asleep), sleep duration, habitual sleep efficiency (*i.e*., the percentage of time in bed that one is asleep), sleep disturbances, use of sleeping medication, and daytime dysfunction. Each item is weighted on a 0–3 interval scale. The global PSQI score is then calculated by totaling the seven component scores, providing an overall score ranging from 0 to 21, where lower scores denote a healthier sleep quality (Buysse et al., 1989).

#### Self-Efficacy Scale:

It consists of 17 items that assess a “general set of expectations that the individual carries into new situations, rated on a five-point scale ranging from “agree strongly” to “disagree strongly”. Higher scores represent higher general self-efficacy (Sherer and Maddux, 1982).

#### Depression, Anxiety and Stress Scale (DASS-21):

This is a set of three self-report scales designed to measure the emotional states of depression, anxiety, and stress. Each of the three DASS-21 scales contains 7items, divided into subscales with similar content. Scores on the DASS-21 are added and multiplied by 2 to calculate the final score (Henry, 1995).

#### Symbol Digit Modalities Test (SDMT):

This is a screening instrument commonly used in clinical and research settings to assess neurological dysfunction. Performance on the SDMT is underpinned by attention, perceptual speed, motor speed, and visual scanning (Smith, 1973).

#### California Verbal Learning Test-II (CVLT-II):

This is an updated version of the original CVLT, which has been standardised and provides normative data. It is one of the most widely used neuropsychological tests. It was designed to not only measure how much a subject learned, but also reveal the strategies employed and the types of errors made. The CVLT indexes free and cued recall, serial position effects (including primacy and recency), semantic clustering, intrusions, interference and recognition (Delis et al., 1987).

#### Brief Visuospatial Memory Test-Revised (BVMT-R):

This is used to evaluate visuospatial memory. The individual is presented with a matrix of six items for ten seconds and asked to replicate the matrix using pencil and paper while taking as much time as needed. The process is repeated three times. Each drawing is evaluated for both its placement (1 point) and its accuracy (1 point). The maximum score yielded by the three trials is 36 (Benedict, 1997).

#### Pythagorean Self Awareness Intervention:

The PSAI technique was delivered by health professionals (FV and CD) with expertise in stress management, who provided weekly guidance to the enrolled caregivers, during group sessions (the PSAI technique was taught during the first session, whereas problems and barriers of PSAI practice were resolved in the ensuing sessions). The PSAI sessions took place once a week and lasted for 8 weeks. Health professionals also administered diaries to the participants to record their PSAI practice at home. The five steps were followed in a precise order twice a day, as briefly outlined in Table 1. To begin, right before bedtime participants need to sit in a quiet and relaxing place and practice diaphragmatic breathing for 5 minutes, to concentrate. Participants are then expected to recall every event that occurred during the day, all their actions, and reactions to feelings they experienced. In the third step, participants need to examine whether they followed a healthy lifestyle during the day or not. In the fourth step, they critically appraise their actions and decide what was done as it should have been done, and what could have been done in a better manner. During the fifth and final step in the following morning, participants summarize their conclusions from the night before and set the goals for the day.

### Statistical analysis

Statistical analysis included simple univariate tests (Pearson’s chi-square test for categorical variables and Student’s t tests for numerical variables). The Cohen’s d effect size was calculated. A per-protocol analysis was conducted, although no dropouts were noted. The level of significance was set at 0.05. The SPSS 21.0 (Chicago IL) software was used for the analyses.

## Results

A total of 59 non-paid caregivers participated in this quasi-experimental study and were assigned to either the PSAI group (n=28) or the control group (n=31). Caregivers of the PSAI group demonstrated full compliance with the intervention. No side-effect was reported by any of the participants. Sociodemographic characteristics of study’s groups are presented in Table 2. No dropouts occurred during the follow-up in either of the groups.

No significant differences were noted between study groups at baseline. Table 3 presents the outcome measures’ differences before and after the intervention for both groups. Significant improvements were found for all outcomes based on the effect size (*i.e*., Cohen’s d).

## Discussion

The aim of the present study was to assess the effects of the novel Pythagorean Self-Awareness Intervention on the stress levels and specific psychological and cognitive functions of caregivers of inpatients with motor disabilities due to chronic neurological diseases. The initial hypothesis that the PSAI would improve caregivers’ psychological dimensions, sleep quality, and cognitive aspects was verified. Although there were significant improvements in all measured characteristics, the strongest effect sizes were observed in stress, anxiety, sleep quality, self-efficacy, and visual memory. In 1990, Pearlin proposed a stress model for caregivers, which divided stressors in primary and secondary, *i.e.* daily activities, role factors (intra-family relationships), behavioural disturbances, social isolation, lack of knowledge about the patient’s disease and economic concerns were included (Pearlin et al., 1990). Positive effects of the PSAI on the stress level could result in improved quality of life. Improvement of anxiety levels is of high importance for the caregivers of patients (Moss et al., 2019); caregivers carry a variety of responsibilities thatrange from managing the patient’s anxiety to performing practical tasks (such as bathing, feeding and dressing of the patient). It is estimated that almost three quarters of a caregiver’s life is dedicated in caregiving (Aguglia et al., 2004), while the reported prevalence of anxiety in caregivers is high, up to 43% (Sallim et al., 2015). Thereby, the strong effect size of the PSAI program on anxiety levels could be extremely beneficial and smoothening for them.

The findings of this study are in line with the results of other studies reporting the positive effects of the PSAI. Charalampopoulou *et al*., investigated the effects of the PSAI on the psychological symptoms of breast cancer patients on cancer treatment, and found a reduction in perceived stress and anxiety symptoms of the patients. Secondary outcomes *i.e*., sleep quality and self-efficacy were also improved after PSAI (Charalampopoulou *et al*., 2020). Encouraging results have been also reported by Simos *et al*. who implemented the PSAI on obese/overweight individuals and found significant effect sizes with respect to perceived stress levels, lifestyle and eating behaviours (Simos et al., 2019).

Many studies have investigated the effects of different stress management methods on caregivers. A meta-analysis of the effects of internet-based interventions for stress reduction of caregivers of dementia patients showed not only positive but also negative effects (Zhao et al., 2019). On the contrary, a systematic review on the effects of cognitive behaviouural therapy for depression, anxiety and stress in caregivers of dementia patients, revealed positive effects on depression and stress, but no significant difference in anxiety (Hopkinson et al., 2019). Another meta-analysis that studied the distress of caregivers of mentally ill patients as a secondary outcome, included studies implementing various techniques, such as psychoeducation, group support and self-management techniques. Overall, all methods showed positive results, but for some of them these results were not maintained in follow-up measurements (Yesufu-Udechuku et al., 2015).

The present study examined the effects of the novel and introspective PSAI. PSAI shares similarities with other cognitive based interventions (CBIs), as it aims to cognitively reconstruct problematic behavioural characteristics and rectify harmful aspects of individuals’ lifestyle. Yet, there are specific elements of the PSAI which could render it more effective and a better choice than other CBIs. Cognitive based interventions usually demand at least few months of over 24 sessions to start bringing about behavioural alterations, as well as the constant presence and guidance of a trained therapist. Therefore, these types of interventions or treatments carry a great financial cost, making them accessible only to a portion of people. The PSAI overcomes these obstacles; individuals need only a short period of training by professionals, who will teach them the theory behind it and can subsequently implement the steps of the intervention on their own, whenever and wherever they wish.

Study limitations include the small sample size that impedes generalization of the results, the lack of randomization and the use of subjective self-report measures that might compromise the validity of results, and the short follow-up period. However, large effect sizes were attested for crucial endpoints concerning caregiving, thus further research is warranted.

The psychosocial burden of caregivers, especially when family-related to patients, may lead to detrimental results such as anxiety, depression, and distress. Various techniques have been used to manage the negative impact on the individuals with caregiving responsibilities. In the small sample studied, the PSAI was promising in reducing stress, anxiety, and depressive symptoms as well as improving sleep quality, self-efficacy, and cognitive processes in caregivers of inpatients with motor disabilities. Future randomized controlled studies are needed to investigate the effects of this novel intervention in larger samples of caregivers.

## Figures and Tables

**Table 1.** Pythagorean Self Awareness Intervention steps.

**Step 1**	Sit at bedside and read the Pythagorean verses
**Step 2**	Recall every event of the day in the sequence they occurred
**Step 3**	Visualise yourself as an observer and contemplate on day’s physical activity, diet, sleep and interpersonal interactions
**Step 4**	Critically appraise each event by asking yourself “In what have I done wrong ?What have I done right ? What have I omitted that I ought to have done?”
**Step 5**	After wake-time, summarize the conclusions from the previous night and set goals for the day

**Table 2.** Sociodemographic characteristics of participants.

Characteristic	PSAI group (n=28)	Control group (n=31)
Age (mean ± SD)	52.2 ± 12.9%	53 ± 13.8%
Females	78.6	87.1
Tertiary education	75	74.2
Married	50	48.4
Employed	75	77.4

PSAI: Pythagorean Self Awareness Intervention, SD: Standard deviation

**Table 3.** Comparisons of outcome change differences between study groups.

Measure	PSAI (N=28)	Controls (N=31)	p value^1^
Stress	−12.5 ± 4.5	−1.0 ± 2.3	<0.0001*
Anxiety	−2.4 ± 2.6	−0.3 ± 1.6	<0.0001*
Depression	−2.9 ± 3.2	0.7 ± 2.0	0.003*
Sleep Quality	−2.4 ± 2.0	0.1 ± 0.6	<0.0001*
Self-efficacy	2.5 ± 2.5	−0.4 ± 1.0	<0.0001*
Cognitive Speed Processing	2.43 ± 3.70	0.90 ± 1.58	0.04*
Verbal Memory	2.32 ± 6.85	0.74 ± 1.98	0.224
Visual Memory	4.3 ± 2.8	0.7 ± 1.5	<0.0001*

*Values represent means* ± *standard deviations. Outcome measures’ differences calculated as baseline minus follow up. 1Student’s t-test*,

* Significant difference at p ≤0.05

PSAI: Pythagorean Self Awareness Intervention

## Key Points

- Pythagorean Self-Awareness Intervention (PSAI) is a novel stress management technique.
- Caregivers of inpatients with motor disabilities practiced the PSAI for 8 weeks.
- PSAI reduced caregivers’ stress, anxiety, and depressive symptoms.
- PSAI improved caregivers’ sleep quality, self-efficacy, and cognitive processes.
